# Lactate Dehydrogenase-Elevating Virus Induces Systemic Lymphocyte Activation via TLR7-Dependent IFNα Responses by Plasmacytoid Dendritic Cells

**DOI:** 10.1371/journal.pone.0006105

**Published:** 2009-07-01

**Authors:** Christoph G. Ammann, Ronald J. Messer, Karin E. Peterson, Kim J. Hasenkrug

**Affiliations:** Laboratory of Persistent Viral Diseases, Rocky Mountain Laboratories, National Institute of Allergy and Infectious Diseases, National Institutes of Health, Hamilton, Montana, United States of America; University of Sao Paulo, Brazil

## Abstract

**Background:**

Lactate dehydrogenase-elevating virus (LDV) is a natural infectious agent of mice. Like several other viruses, LDV causes widespread and very rapid but transient activation of both B cells and T cells in lymphoid tissues and the blood. The mechanism of this activation has not been fully described and is the focus of the current studies.

**Principal Findings:**

A known inducer of early lymphocyte activation is IFNα, a cytokine strongly induced by LDV infection. Neutralization of IFNα in the plasma from infected mice ablated its ability to activate lymphocytes *in vitro*. Since the primary source of virus-induced IFNα *in vivo* is often plasmacytoid dendritic cells (pDC's), we depleted these cells prior to LDV infection and tested for lymphocyte activation. Depletion of pDC's *in vivo* eradicated both the LDV-induced IFNα response and lymphocyte activation. A primary receptor in pDC's for single stranded RNA viruses such as LDV is the toll-like receptor 7 (TLR7) pattern recognition receptor. Infection of TLR7-knockout mice revealed that both the IFNα response and lymphocyte activation were dependent on TLR7 signaling *in vivo*. Interestingly, virus levels in both TLR7 knockout mice and pDC-depleted mice were indistinguishable from controls indicating that LDV is largely resistant to the systemic IFNα response.

**Conclusion:**

Results indicate that LDV-induced activation of lymphocytes is due to recognition of LDV nucleic acid by TLR7 pattern recognition receptors in pDC's that respond with a lymphocyte-inducing IFNα response.

## Introduction

Lactate dehydrogenase-elevating virus (LDV) is a small, positive sense, single stranded RNA virus of the Arteriviridae family, related to coronaviruses such as the severe acute respiratory syndrome (SARS) virus [1], [2], [3], [4]. It is a natural infectious agent of mice that causes very rapid lytic infections generally restricted to a minor subset of non-essential macrophages involved in scavenging extracellular lactate dehydrogenase [5], [6]. The rapid loss of this subset results in the elevated lactate dehydrogenase levels for which the virus is named [7]. Virus titers peak within the first day of infection as susceptible target cells are depleted, and then the infection is maintained at a much lower chronic level dependent on the replenishment of new macrophage targets [8]. LDV establishes chronic infections regardless of mouse strain, age, sex or immune-status [5], [8], [9], [10]. No clinical signs are typically associated with LDV infections, although co-infection with retroviruses can lead to CNS disease under certain circumstances [11], [12], and mice infected with LDV have suppressed immune responses [13], [14], [15], [16]. We recently found that acute infection with LDV induced a state of partial and transient activation in the vast majority of splenic lymphocytes. Activation was characterized by high surface expression of the very early activation marker CD69 [16]. CD69 is the first surface marker known to be upregulated during the activation of lymphocytes and has recently been shown to interact with S1P1 to inhibit the egress of lymphocytes from lymphoid tissues [17]. CD69 expression is upregulated by T cell receptor (TCR) ligation [18] but is not dependent upon it and can be induced by inflammatory cytokines such as IFNα [19], [20].

## Results

**Figure 1:**
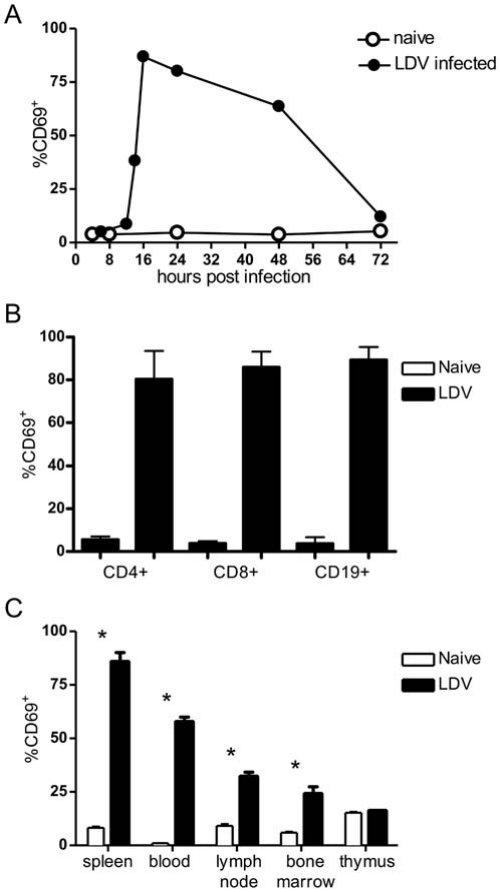
LDV-induced upregulation of CD69. Cells were stained and analyzed by flow cytometry as described (22). (A) Kinetics showing the percentage of splenic lymphocytes with CD69 upregulation. Significant upregulation (P<.05) was observed between 12 and 48hpi (n = 2−4 mice per group per time point). (B) Upregulation of CD69 on major splenic lymphocyte subsets at 16hpi with LDV was analyzed by co-staining with antibodies for CD69 and either CD4, CD8 or CD19 as indicated. The difference in percentage of CD69^hi^ cells between naïve and infected mice was statistically significant for all subsets by t test, P = 0.0225 for CD4^+^ T cells, 0.003 for CD8^+^ T cells, and 0.0018 for CD19^+^ B cells (n = 2 mice per naïve group and 3 mice per infected group.) (C) Tissue distribution of CD69 upregulation. Lymphocytes from spleen, blood, lymph nodes, bone marrow, and thymus were examined at 16hpi with LDV. All tissues except the thymus showed a significantly higher percentage of CD69^hi^ lymphocytes when infected with LDV infected (P<0.01 for all groups indicated by *).

To investigate the mechanism of lymphocyte activation following LDV infection we first analyzed the kinetics of CD69 upregulation on splenic lymphocytes at several time points following infection. CD69 expression was analyzed by flow cytometry as previously described [16] and became detectable at 14 hours post-infection, peaked at 16 to 24 hours, and returned almost to background levels by 72 hours (Figure 1A). The induction of CD69 occurred on CD4+ and CD8+ T cells, as well as B cells (Figure 1B). In addition to the spleen, CD69 upregulation was also observed, albeit to a lesser extent, on lymphocytes from the blood, lymph nodes, and bone marrow (Figure 1C). In contrast, no significant upregulation was observed on lymphocytes from the thymus, which are primarily immature T cells. In contrast to CD69, the IL-2 receptor alpha chain (CD25), which is upregulated later in the activation cascade and is typically dependent on TCR ligation [21], did not increase in expression during the first day of LDV infection (data not shown). This result is consistent with partial rather than full activation of the lymphocytes.

**Figure 2:**
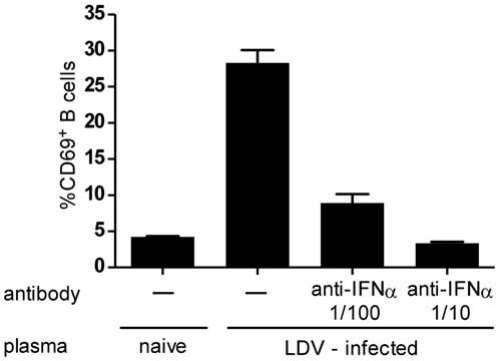
CD69 upregulation in B cells blocked by anti-IFNα antibody. The addition of 10% serum from LDV-infected mice harvested at 16hpi induced upregulation of CD69 in a significant percentage of splenic B cells (2×10^5^ B cells/well) cultured *in vitro* for 4 hours following their isolation with CD19 magnetic beads (Miltenyi Biotec). Addition of 2 or 20 µl of anti-IFNα antibody (PBL Interferon Source) to 198 or 180 µl cultures respectively, significantly reduced the percentage of cells with CD69 upregulation (P≤0.0001 by t test).

The rapid systemic appearance of CD69 expression suggested that a soluble factor such as IFNα, a known early responder to viral infections [22] and strong trigger of CD69 expression [17], [19], [20], was inducing the response. To determine whether the LDV-induced IFNα response [16] could be responsible for CD69 induction, we first utilized the fact that IFNα induces CD69 expression on B cells *in vitro* [19]. Splenic B cells were isolated from naïve mice using CD19+ magnetic beads (Miltenyi Biotec) and cultured with 10% plasma taken from mice infected 16 hours earlier with LDV. B cells cultured for 4 hours with plasma from infected, but not uninfected mice, significantly upregulated CD69 expression (Figure 2). Furthermore, upregulation of CD69 expression was prevented by addition of a neutralizing antibody specific for IFNα (PBL Interferon Source) in a concentration-dependent manner. These findings suggested that the IFNα response to LDV infection might be responsible for the partial activation of lymphocytes *in vivo* as well.

**Figure 3:**
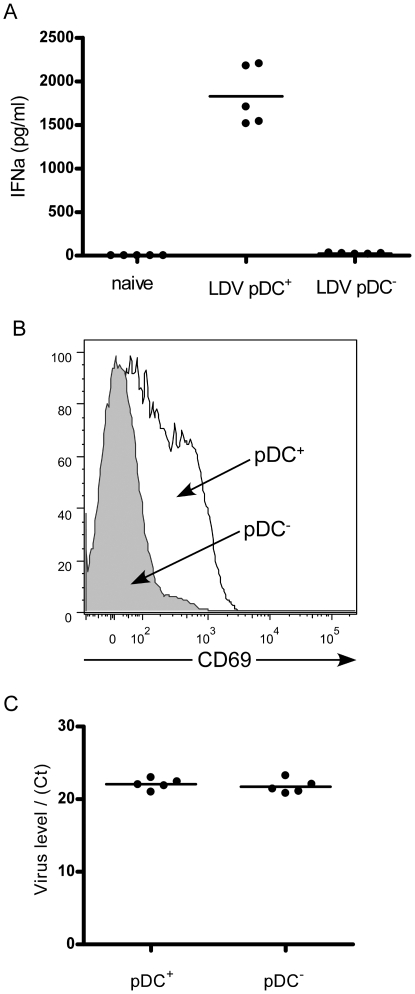
*In vivo* depletion of plasmacytoid dendritic cells abolishes IFNα production. Naïve (A.BY x B10)F1 mice were depleted of pDC's by injection of 0.5 mg mPDCA-1 antibody (Miltenyi Biotec) 24 h prior to infection with LDV, and blood samples were collected at 16hpi. (A) Interferon alpha levels in naïve mice (left), LDV-infected normal mice (middle), and LDV-infected, pDC-depleted mice (right) were measured by ELISA (PBL Interferon Source). The difference between the two LDV-infected groups was statistically significant by t test (P<0.0001, n = 5). (B) Flow cytometric analysis of peripheral blood lymphocytes showed significant reductions in LDV-induced expression of CD69 in pDC depleted mice (average mean fluorescence intensity of 114.3+/−14.25 vs. 42.96+/−11.85, n = 5, p = 0.0049 by t test). A representative histogram of CD69 expression on cells from LDV-infected mice that were pDC-depleted pDC^−^) or mock-treated (pDC^+^) is shown. The histograms of cells from naïve animals overlapped with the curves from pDC depleted mice (data not shown). (C) pDC depletion did not significantly alter LDV levels in plasma as indicated by results from semiquantitative real-time RT-PCR as previously described [16]. Relative LDV levels are indicated by real time PCR critical threshold (Ct) values, which were not significantly different between LDV-infected normal mice (pDC^+^) Ct = 22.05+/−0.33, and LDV-infected, pDC-depleted mice (pDC^−^) Ct = 21.72+/−0.43 (n = 5).

Although any cell can produce IFNα in response to infection, the acute systemic response to viruses has been attributed to production by plasmacytoid dendritic cells (pDC's, also known as interferon-producing cells or IPC) [23], [24], [25], which comprise only a minor subpopulation of cells but can produce 1000 times as much IFNα as other cells [24]. Conventional DC's can also produce high amounts of IFNα if they are directly infected, but pDC's are uniquely able to secrete high levels of IFNα in response to endocytosed antigen. The role of pDC's in production of IFNα during LDV infection was investigated by depleting mice of pDC's the day before LDV infection using a pDC-specific depleting antibody [26]. The plasma IFNα response at 16 hours post-infection with LDV, as measured by ELISA, was abolished by pDC depletion (Figure 3A). Thus the systemic IFNα response was predominantly due to production by pDC's. In addition to loss of the IFNα response in pDC-depleted mice, we also observed the failure of splenic lymphocytes to upregulate CD69. A histogram showing CD69 expression on splenocytes from a representative mouse is shown in Figure 3B. Combined with the dependence on IFNαfor upregulation of CD69 on B cells *in vitro*, the data indicate that *in vivo* upregulation of CD69 on lymphocytes is likely due to the systemic IFNα response to LDV infection. Interestingly, the loss of the IFNα response in pDC-depleted mice produced no statistically significant difference in LDV plasma levels as measured by real time PCR (15) (Figure 3C). Since IFNα can act in both autocrine and paracrine manners to limit virus replication and spread [27], it appears that LDV is quite resistant to the antiviral effects of IFNα, even when present at very high systemic levels.

**Figure 4:**
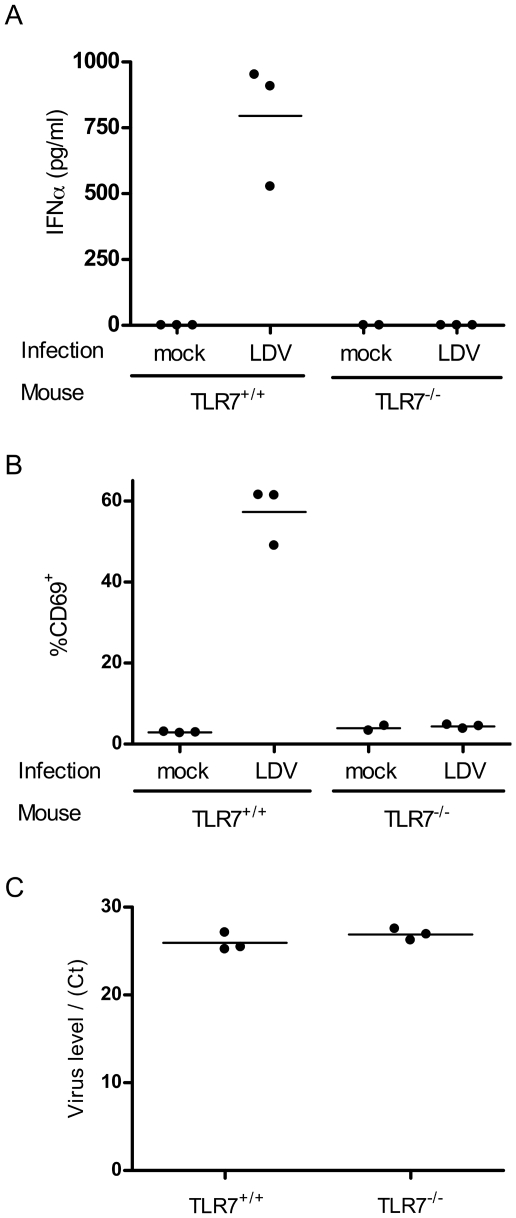
LDV-induced CD69 upregulation is TLR7-dependent. TLR7 wild type and knockout mice on the 129SvEv genetic background were infected with LDV and blood was analyzed at 16hpi. (A) IFNα levels in plasma were significantly reduced in TLR7^−/−^ mice as measured by ELISA (PBL Interferon Source). Values in TLR7^+/+^ mice averaged 795.5 pg/ml while only trace amounts of IFNα were detected in TLR7^−/−^ mice ( TLR7^+/+^ vs. TLR7^−/−^ p = 0.0041 by T test). (B) The low levels of IFNα in TLR7^−/−^ mice correlated with reduced percentages of lymphocytes expressing CD69 (TLR7^+/+^ 57.30%+/−4.15% CD69^+^ vs. TLR7^−/−^ 4.37%+/−0.28% CD69^+^, n = 3, p = 0.0002 by t test). (C) LDV-specific semiquantitative real-time RT-PCR as previously described [16] revealed no significant difference in virus levels between TLR7 knockout and TLR7 wild type mice as measured by critical threshold values (TLR7^+/+^ Ct = 25.93+/−0.59 vs. TLR7^−/−^ 26.90+/−0.38, n = 3 mice/group).

Given that LDV is a single-stranded RNA virus, we investigated whether the pDC-dependent IFNα response was mediated by toll like receptor 7 (TLR7), which is highly expressed by pDC's, binds to single stranded viral RNA, and is capable of initiating IFNα responses in pDC's without their direct infection [28]. Mice containing a genetically inactivated TLR7 gene [29], [30] failed to mount IFNα responses or to upregulate CD69 expression in response to LDV infection, whereas genetically matched TLR7 wild type mice showed strong IFNα responses and CD69 upregulation (Figure 4A, B). Consistent with the results from pDC depletions, LDV plasma titers were again not significantly different in the absence of TLR7 expression and IFNα production (Figure 4C). These results are similar to data from type I interferon receptor-deficient mice infected with LDV, although that study noted slight (two fold) increases in virus titers in the absence of type I interferon signaling [31].

## Discussion

Together, our data indicate that pDC's activated in a TLR7-dependent manner are primarily responsible for the rapid systemic IFNα response following infection of mice with LDV. Furthermore, the interferon response was most likely responsible for the transient expression of the CD69 very early activation marker on lymphocytes during acute LDV infection. IFNα–dependent, partial activation of lymphocytes has also been reported during acute infection with Semliki forest virus [20], human adenovirus 2, West Nile virus, and A/WSN influenza virus [32] in mice. However, not all acute viral infections induce partial activation, as it does not occur in Friend retrovirus infections of mice [16]. Such broad activation is by definition non-specific, and leaves open the question of how it benefits the host. Alsharifi et al. have proposed that IFNα-induced partial activation may promote adaptive immune responses by lowering the threshold for full activation once antigen-specific recognition occurs [33]. If so, IFNα may be a very important regulatory link between the innate and adaptive immune responses. Based on the findings that CD69 acts downstream of IFNα to inhibit lymphocyte egress from lymphoid organs [17], it is also likely that CD69 expression facilitates sustained contacts between lymphocytes and antigen presenting cells during inflammatory responses, thereby enhancing full activation of antigen-specific lymphocytes.
